# Combining TGF-β1 knockdown and miR200c administration to optimize antitumor efficacy of B16F10/GPI-IL-21 vaccine

**DOI:** 10.18632/oncotarget.3722

**Published:** 2015-03-30

**Authors:** Xiaoying Wang, Fengshu Zhao, Xiangfeng He, Jing Wang, Ying Zhang, Hongyi Zhang, Yaoyao Ni, Jianan Sun, Xiaobing Wang, Jun Dou

**Affiliations:** ^1^ Department of Pathogenic Biology and Immunology, School of Medicine & Collaborative Innovation Center of Suzhou Nano Science and Technology, Southeast University, Nanjing, China; ^2^ Department of Medical Oncology, Affiliated Tumor Hospital of Nantong University, Nantong, China; ^3^ Department of Gynecology and Obstetrics, Zhongda Hospital, School of Medicine, Southeast University, Nanjing, China; ^4^ Department of Center for Experiment Animal, School of Medicine, Southeast University, Nanjing, China

**Keywords:** melanoma, tumor vaccine, interleukin-21, transforming growth factor β1, miR200c

## Abstract

TGF-β1 secreted abundantly by tumors cells as well as present in the local microenvironment promotes neoplasm invasion and metastasis by triggering the epithelial to mesenchymal transition (EMT). MiR200c has been shown to suppress EMT and to regulate the cellular epithelial and interstitial state conversion, whereas the tumor vaccines are intended to specifically initiate or amplify a host response against evolving tumor cells. Our study aimed at optimizing the antitumor effects of the B16F10/glycosylphosphatidylinositol-interleukin 21 (B16F10/GPI-IL-21) tumor vaccine on melanoma bearing mice by combining the TGF-β1 knockdown and the administration of miR200c agomir. The mice were subcutaneously vaccinated with inactivated B16F10/GPI-IL-21 vaccine and challenged by B16F10 cells transfected with shTGF-β1 (B16F10/shTGF-β1 cells) or B16F10/shTGF-β1 cells with the administration of miR200c agomir. The later combination showed that, when compared with the mice in the control group that received no vaccination, vaccinated mice significantly increased NK and CTL activities, enhanced levels of IFN-γ, and reduced expression of TGF-β1, N-cadherin, Vimentin, Gli1/2, P-Smad2/3 and others involved in promoting expression of EMT-related molecules in tumor areas, and inhibited the melanoma metastasis in lungs and lymph nodes. Altogether, our findings demonstrate that this synergistic anti-cancer regimen effectively induces strong immune response and diminishes the melanoma progression.

## INTRODUCTION

When melanoma spreads throughout human body, however, there is currently no reliable therapy for advanced malignant melanoma. Both the underlying the molecular mechanisms of the progression of the disease and the effective strategies for the treatment of advanced melanoma remain inadequately defined [1-3].

Accumulating evidence supports the claim that the tumor cell-based therapeutic vaccines may be employed to specifically initiate or amplify a host immune response against evolving tumor cells [4-6]. We have previously reported that the tumor vaccine of the glycosylphosphatidylinositol (GPI)-anchored interleukin 21(B16F10/GPI-IL-21) in combination with the overexpression of microRNA-200c (miR200c) or with a knockdown of the zinc-finger E-box binding homeobox 1(ZEB1), which is a transcription factor [7], significantly induced immune responses to the B16F10 cells, diminished tumor growth and inhibited the melanoma epithelial to mesenchymal transition (EMT) program. However, this tumor vaccine failed to completely eliminate malignant B16F10 melanoma or prevented lung metastases in a murine melanoma model because the mice developed measurable and metastatic tumors. This failure stresses the need for designing a novel anti-cancer regimen to induce a strong antitumor immunity and modify melanoma's properties for inhibiting tumor metastasis [8, 9].

Previous studies have found that molecules such as miR200c, ZEB1 and transforming growth factor β (TGF-β), can potentially regulate tumor progression [4, 10, 11], and that the overexpression of miR200c or the knockdown of TGF-β1 or ZEB1 could inhibit the tumor cell EMT program that activates cellular mobility and subsequent tumor metastasis [12-14], in particular, the TGF-β1 signaling blockade has proven to be efficient in preventing the development of a variety of tumor types [15]. Rapid progression of primary melanomas to locally invasive and metastatic ones remains a major obstacle to the cure of melanoma in patients. Recent significant progress in the melanoma research affords us to hypothesizing that antitumor immune responses initiated by the tumor vaccine B16F10/GPI-IL-21 might lead to the suppression of the B16F10 cell proliferation or apoptosis, and that this, however, does not completely block the B16F10 cell metastasis in mice [7, 8]. On the other hand, the EMT-inducer TGF-β1 may promote tumor metastasis in B16F10 melanoma-bearing mice through increased tumor cell mobility and dissemination. For this reason, we designed an anti-melanoma paradigm that employed the down-regulation of TGF-β1 in combination with the injection of miR200c agomir to enhance the antitumor effect of the B16F10/GPI-IL-21 vaccine on the B16F10 melanoma bearing mice. We demonstrate that this combination significantly repressed tumor growth, blocked the melanoma EMT program and inhibited tumor metastasis in the mice by inducting strong immune responses and inhibiting the TGF-β signal pathway.

## RESULTS

### EMT-associated molecule expression and analysis of cell biological property in B16F10/shTGF-β1 cells

**Figure 1:**
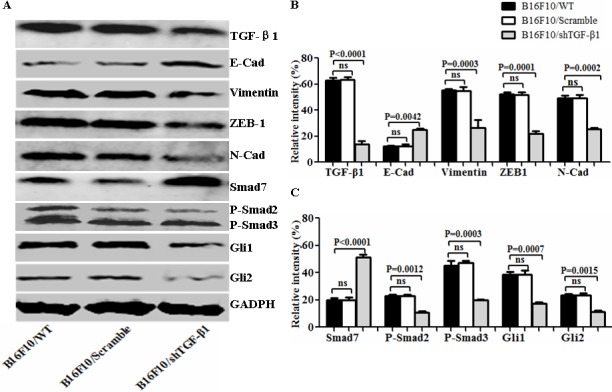
Analysis of EMT-associated molecule expression in differently treated B16F10 cells **A**. EMT associated molecular expression analyzed by Western blot in B16F10/wild type (WT), B16F10/Scramble, and the B16F10/shTGF-β1 cells. **B** and **C**. Semi-quantification analysis of relative intensity of molecular expression; refer to the statistical differences as indicated. Relative intensity of GADPH expression was served as the 100%, and other molecular expression was compared with GADPH expression.

**Figure 2:**
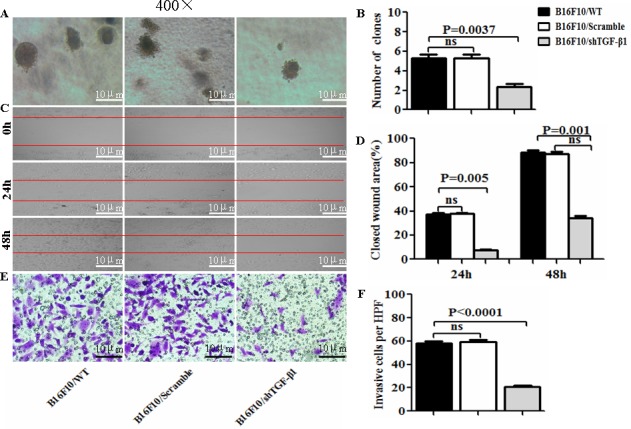
Observation of cell biological property in B16F10/shTGF-β1 cells **A**, **C** and **E**. The images of colony forming rates, of the cellular wound areas, and of the invasive cells were taken from B16F10/WT, B16F10/Scramble, and the B16F10/shTGF-β1 cells, respectively. **B**, **D** and **F**. Statistical analyses of the colony forming rates, the cellular wound areas, and the invasive cells per high power field (HPF, ×400) respectively. The data are represented as mean +/− SEM (n=6); refer to the statistical differences as shown.

To evaluate the influence of the TGF-β1 knockdown on the B16F10 cells, we first developed an expression vector-based small hairpin RNA to target TGF-β1 (shTGF-β1) and then transfected it into the B16F10 cells. In terms of the intercellular regulation of TGF-β1, we found that the TGF-β1 expression was significantly decreased in the B16F10/shTGF-β1 cells compared with the B16F10 cells and the B16F10/Scramble cells (Figure 1A and 1B). The EMT associated molecular expression of E-cadherin and Smad 7 was obviously increased whereas the expression of N-cadherin, Vimentin, Gli1, Gli2, P-Smad2, and P-Smad3 such as those involved in the EMT-related molecules were markedly decreased in the B16F10/shTGF-β1 cells in comparison with the B16F10 cells and the B16F10/Scramble cells (Figure 1). Next, we analyzed the properties of the B16F10/shTGF-β1 cells by observation of the clone formation, and cellular invasive and metastatic ability *in vitro*. The down-regulation of the TGF-β1 expression in the B16F10/shTGF-β1 cells significantly reduced the colony forming rates and the cellular invasive and metastatic ability in comparison with the B16F10 cells and the B16F10/Scramble cells; the comparisons are shown in Figure 2A, 2C and 2E, respectively. The statistical analysis results in Figure 2B, 2D and 2F show a significant differences between the B16F10/shTGF-β1 cells and the B16F10 cells or the B16F10/Scramble cells. These results suggested that the shTGF-β1 transfected B16F10 cells demonstrated the potential for inhibiting cellular proliferation potential and EMT property.

### Induced immune responses in vaccinated mice challenged with B16F10/shTGF-β1 cells plus minus miR200c agomir

**Figure 3:**
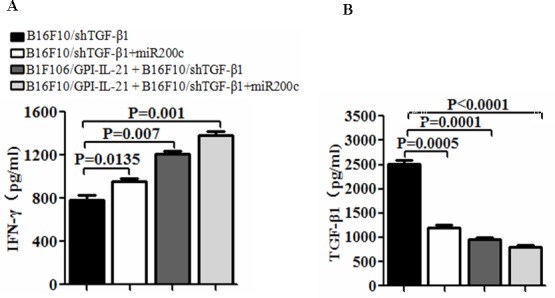
Serum levels of IFN-γ and TGF-β1 in vaccinated mice challenged with B16F10/shTGF-β1 cells **A** and **B**. Serum levels of IFN-γ and TGF-β1 were tested by enzyme linked immunosorbent assay. The mice were first immunized s.c. with the B16F10/GPI-IL-21 inactivated tumor vaccine and then were challenged by the differently treated B16F10 cells as described in the section of materials and methods. Data are represented as mean +/− SEM (n=12).

To evaluate the immune efficacy of the B16F10/GPI-IL-21 vaccine, we first measured the cytokine IL-21 expression. The results from the Western blot indicate that IL-21 was expressed in the B16F10/IL-21-GPI cells; this was further confirmed by the immunofluorescence assay as was previously reported (data not shown here) [9]. Next, we measured serum cytokine levels in the mice that received different treatments. Figure 3A and 3B show that the serum IFN-γ level was significantly elevated, whereas the TGF-β1 level was notably decreased in the mice that were immunized with B16F10/GPI-IL-21 and challenged by B16F10/shTGF-β1 cells with miR200c agomir injection, or the B16F10/GPI-IL-21 immunized mice challenged by the B16F10/shTGF-β1 cells, in comparison with the non-vaccinated mice that were challenged by the B16F10/shTGF-β1 cells with injection of miR200c or challenged by the B16F10/shTGF-β1 cells only. The differences were statistically significant.

**Figure 4:**
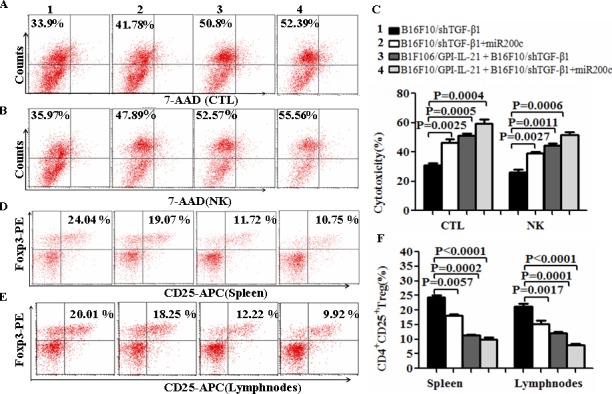
The activities of CTL and NK and CD4**^+^**CD25^+^Treg cell level in vaccinated mice challenged with B16F10/shTGF-β1 cells **A** and **B**. The CTL and NK cytotoxic activities were analyzed by Flow Cytometry for the differently treated groups, and the quantification analysis was shown in **C**, **D** and **E**. The level of CD4^+^CD25^+^Treg cells was analyzed by a Flow Cytometry in splenocytes (D) and tumor draining lymph nodes (E); refer to the statistical differences as shown in F. n=9.

Further, we evaluated the splenocyte cytotoxicity to the B16F10 cells or to the YAC-1 cells, which represents to the CTL or NK activity. The results showed that the B16F10/GPI-IL-21 combined with the down-regulated TGF-β1 and the administration of miR200c agomir group remarkably enhanced the CTL and NK cytotoxic activities in comparison with any other groups (Figure 4A and 4B); the differences were statistically significant (Figure 4C). In addition, the CD4^+^CD25^+^Treg cells derived from the splenocytes and tumor draining lymph nodes were significantly reduced in the tumor vaccine in combination with shTGF-β1 and miR200c group as shown in Figure 4D, 4E and 4F; these findings suggested that the multiple immune effects were induced through using the tumor vaccine B16F10/GPI-IL-21 combined with the down- regulated TGF-β1 and the administration of miR200c agomir.

### Inhibition of melanoma growth and metastasis in vaccinated mice challenged with B16F10/shTGF-β1 cells plus minus miR200c agomir

**Figure 5:**
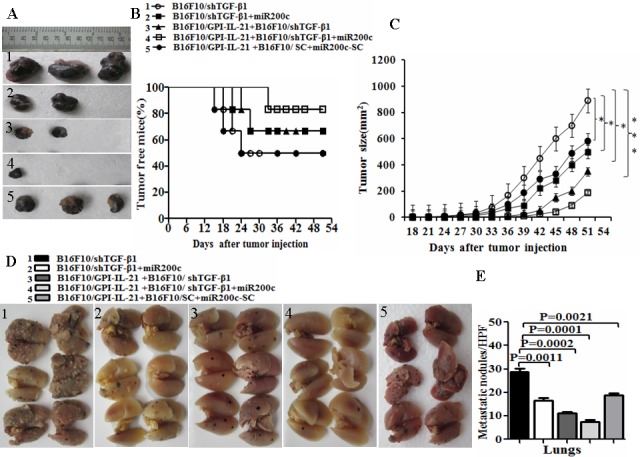
Tumor growth and metastasis in vaccinated mice challenged with B16F10/shTGF-β1 cells plus minus miR200c agomir **A**. Images shows the tumor sizes dissected from the differently treated mice on Day 51. After the mice were initially immunized s.c. with 1×10^6^ B16F10/GPI-IL-21 inactivated tumor vaccine the mice were challenged by the differently treated B16F10 cells as described in the section of materials and methods. **B**. The quantification analysis of tumor sizes (*p < 0.05, ** *p* < 0.01 and *** *p* < 0.001). **C**. Tumor free mice during 51 days. **D**. Images of tumor metastatic focus in mouse lungs. E. The quantification analysis of lung metastatic focus counts (HPF, ×400). Data are represented as mean +/− SEM; refer to the statistical differences as indicated.

To reinforce the antitumor efficacy of B16F10/GPI-IL-21 in the B16F10 melanoma-bearing C57BL/6 mice, we down regulated the TGF-β1 expression in the B16F10 cells by the RNAi technology and injection of miR200c agomir simultaneously. Figure 5A portrays the sizes of tumor dissected from the mice challenged by the differently treated B16F10 cells. We found that 3 of the 6 mice developed tumors on Day 18, Day 21 and Day 24, respectively after the mice were challenged by the B16F10-shTGF-β1 cells, and that 2 of the 6 mice challenged by the B16F10-shTGF-β1 cells with the injection of miR200c agomir developed tumors on Day 21 and Day 27, respectively (Figure 5B). Figure 5A-5B also show that the B16F10/GPI-IL-21 immunized mice developed tumors on Day 24 and Day 27, respectively, after the 6 mice were challenged by the B16F10-shTGF-β1 cells. In contrast, only 1 of the 6 mice in the experiment group grew the smallest tumor on Day 33 (Figure 5A-5C) along with the obvious reduction in pulmonary metastasis (Figure 5D) in the group challenged by B16F10/GPI-IL-21 combined with B16F10-shTGF-β1 and miR200c. No measurable tumors were detected in the remaining 5 mice until 51 days into the observation (Figure 5C).

**Figure 6:**
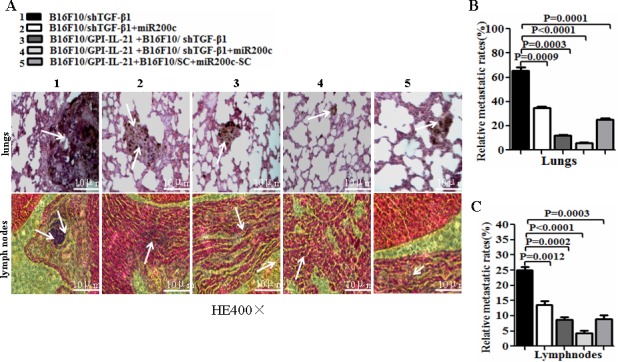
Histological analysis of lungs and lymph nodes of melanoma bearing mice **A**. Tissue sections derived from melanoma bearing mice 51 days after vaccination followed by challenge with the differently treated cells. The top and bottom panels show the lung sections and the lymph node sections, respectively. The arrows point to the metastatic focus as described in the text. **B** and **C**. Quantitative analysis of the tumor metastatic rates of lungs and lymph nodes, respectively, in the differently treated mice; refer to the statistical differences as indicated.

The results from the tumorigenicity and the tumor lung metastasis counts suggested that the synergism antitumor efficacy was found in the mice immunized with the tumor vaccine B16F10/GPI-IL-21 and then challenged by the B16F10-shTGF-β1 cells and the injection of miR200c agomir (Figure 5). Also, these findings were supported by the histological pathologic analysis of the different sections derived from the mice. Figure 6A portrays the apparent metastatic focus seen in the lungs and lymph nodes of the mice challenged by B16F10/shTGF-β1 or challenged by B16F10/shTGF-β1 with the injection of miR200c agomir, even in the B16F10/GPI-IL-21 vaccinated mice with the B16F10/shTGF-β1 challenge. We also found no tumor metastatic focus in the livers, kidneys, and hearts of the experiment mice until 51 days into the observation (data not shown here).

### EMT-related molecular expression in the tumor tissues

**Figure 7:**
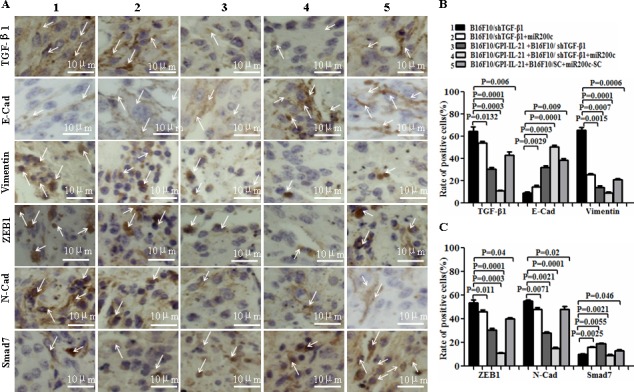

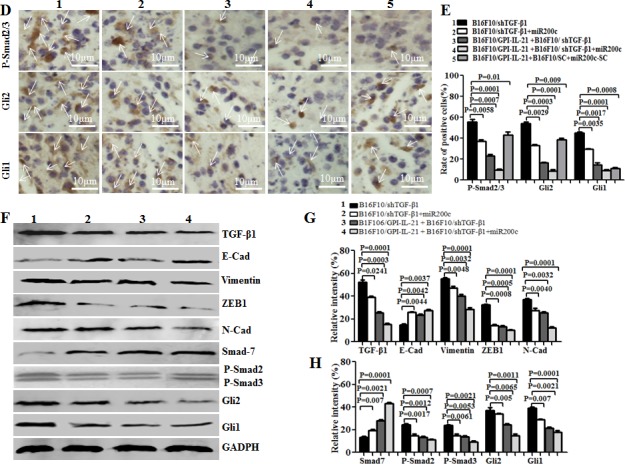
EMT-associated molecule expression analyzed by immu­nohistochemistry and Western blot in vaccinated mice challenged with B16F10/shTGF-β1 cells plus minus miR200c agomir **A** and **D**. Images shows the immu­nohistochemistry results. The arrows point to the EMT associated molecular expressions. **B**, **C** and **E**. Statistical analysis of the positive cells expressed with EMT associated molecules. **F**. EMT associated molecular expression analyzed by the Western blot assay. G and H. Semi-quantification analysis of molecular expression; refer to the statistical differences as indicated.

EMT is a process that exerts influence on many molecular, such as TGF-β, E-cadherin, N-cadherin Vimentin, ZEB-1, SMAD-2, SMAD-3, SMAD-7, GLI1 and GLI2; these molecules are closely associated with typical phenotype changes of EMT in tumor cell growth and metastasis [10, 16-18]. To understand the pathophysiological mechanisms of the above- mentioned antimelanoma efficacy and inhibiting melanoma metastasis in mouse model, we investigated the EMT-related molecular expression in the tumor tissues from the mice that received different treatments. Panels A and D of Figure 7 show the results from the immu­nohistochemistry. Compared with those from other groups, the expression of TGF-β1, Vimentin, ZEB-1, N-cadherin, Gli1/2, and P-Smad2/3 was significantly decreased in the tumor areas from the mice vaccinated with B16F10/GPI-IL-21 combined with the B16F10/shTGF-β1 challenge and the administration of miR200c agomir. Specifically, in panels D, a Gli1 molecule and a GLI2 molecule had weak expressions; the expressions of E-cadherin and SMAD-7 were significantly increased in the tumor areas, and the differences were statistically significant (Figure 7B-7C and 7E). Similarly, the Western blot analysis indicated that the expressions of TGF-β, N-cadherin Vimentin, ZEB-1, SMAD-2, SMAD-3, SMAD-7, GLI1 and GLI2 were all decreased, too, and that the SMAD-7 and E-cadherin expression were markedly increased in the tumor tissues from the mice vaccinated with the B16F10/GPI-IL-21 vaccine and challenged by the B16F10/shTGF-β1 and the injection of miR200c agomir. These results demonstrated that the high expressions of SMAD-7 and E-cadherin, coupled with the low expression of TGF-β, N-cadherin, Vimentin, ZEB-1, SMAD-2, SMAD-3, GLI1 and GLI2, may have inhibited the EMT process of the B16F10 cells.

## DISCUSSION

In this study, we developed a new strategy that combined the B16F10/GPI-IL-21 vaccination with the TGF-β1 knockdown in the B16F10 cells and with the miR200c agomir injection to elicit immune responses against the B16F10 melanoma and to inhibit tumor metastases in a murine model. We first demonstrated that, upon the transfection with recombinant shTGF-β1, the B16F10 cells decreased their proliferation potential, EMT property, and tumor formation, which was reflected in the weak clone formation, the low expression of promoting EMT related molecules, and the decreased cell motility ability *in vitro* as well as the mild tumorigenicity *in vivo*. These findings indicated that the B16F10/shTGF-β1 cells decreased their malignancy.

Investigation of the molecular mechanisms of B16F10/GPI-IL-21 vaccine combined with the TGF-β1 knockdown in the B16F10 cells revealed that this combination caused the mice to develop a significant splenocyte cytotoxicity to the B16F10 cells or the YAC-1 cells; this suggested strong CTL and NK activities, whereas the CD4^+^CD25^+^Treg cells, which are a natural regulatory T cells with repressors of protective immunity and antitumor effectiveness, in splenocytes and tumor draining lymph nodes were obviously decreased. In particular, the mice that received the B16F10/GPI-IL-21 vaccination combined with the B16F10/shTGF-β1 challenge and the administration of miR200c agomir showed stronger CTL and NK activities and lower CD4^+^CD25^+^Treg cells than any other mice; these treated mice also showed a reduction in the serum TGF-β1 level and an increase in the IFN-γ level. It has been reported that IFN-γ can react to NK cells and CTL, enhance their cytotoxic activities, and play a key biological role in killing tumor cells or inducing tumor cell apoptosis [19-21]. Malignant melanomas secrete high amounts of TGF-β, and the increased circulating plasma concentration is asso­ciated with the advanced stage of the tumor. TGF-β also has paracrine effects on angiogenesis and immune surveillance, thereby promoting tumor growth and survival [7, 16, 22, 23]. Our data demonstrated that high level of IFN-γ and low level of TGF-β1 did reinforce antitumor effectiveness and result in being nearly free of the B16F10 melanoma as well as less metastatic focus in the lungs and lymph nodes in the mice that were received B16F10/GPI-IL-21 vaccine and challenged by the shTGF-β1-B16F10 cells along with the miR200c agomir administration.

The GLI2 transcription factor is known to be that often overexpressed in cancers and contrib­ute to the progression of a variety of neoplasms by regulating the cell cycle progression and apoptosis; it is also known that GLI2 is directly induced by TGF-β and SMAD signaling [23, 24]. The GLI1 protein, which is a direct target of GLI2, could induce EMT in the kidney epithelial cells of rat via induction of SNAIL, an E-cadherin repressor [25]. The TGF-β1 signal via membrane-bound heteromeric serine-threonine kinase receptor complexes leads to phosphorylation of proteins of the SMAD family. Phosphorylate SMAD2 and SMAD3 accumulate in the nucleus and act as transcription factors to regulate target gene expression [26, 27]. In this study, we further investigated the roles of TGF-β1 signaling and SMAD- dependent transcriptional re­sponses in the B16F10 cell tumorigenicity and metastasis. Our findings indicated that the reduction of TGF-β1 expression with specific shRNA vector resulted in decreased expression of GLI2, GLI1, phosphorylate SMAD2 and SMAD3 as well as increased SMAD7 expression in the B16F10 cells. Past studies have demonstrated that IL-21 promotes secretion of IFN-γ that induces the expression of Smad7, an antagonistic SMAD, which prevents the interaction of Smad3 with the TGF-β receptor. On the other hand, the enhanced IFN- level inhibited the TGF-β1-induced phosphorylation of Smad3 and its attendant events, namely, the association of Smad3 with Smad4, and the accumulation of Smad3 in the nucleus [8, 28]. Our data showed that the reduction in the GLI2 expression *in vitro* the B16F10/shTGF-β1 cells and *in vivo* tumor tissues may decrease the TGF-β1 target gene expression and may inhibit the pro-oncogenic activity downstream of the TGF-β signaling. These findings revealed the mechanisms of transmodulation between the TGF-β1 and SMAD signal-transduction pathways.

In addition, MiR-200c has been shown to suppress EMT, to attribute to the targeting of ZEB1/ZEB2, repressors of the cell-cell contact protein E-cadherin, and to mainly regulate the cellular epithelial and interstitial state conversion to finish the typical phenotype changes of EMT in the process of tumor cell growth [10, 17, 18]. Therefore, the feasibility and the efficacy of local administration of miR200c agomir augmented the B16F10/GPI-IL-21 vaccine against B16F10/shTGF-β1 cell challenge in our current investigation.

In conclusion, our study indicated that the enhanced antitumor immune response induced by the B16F10/GPI-IL-21 vaccine and the changed EMT-related molecular expression mediated by TGF-β1 knockdown and miR200c agomir optimized the synergism antitumor regimen in the B16F10 melanoma-bearing C57BL/6 mice. This comprehensive efficacy decreased the malignant B16F10 cell's tumor initiation and high metastasis potential in the mice, suggesting that the TGF-β1 signaling blockade and modulation of miR200c impacted cell metastasis by regulating several EMT-related processes. These findings supported the use of a combination of the B16F10/GPI-IL-21 vaccine and the transcriptional regulation of TGF-β1 and miR200c approaches in the clinic melanoma treatment.

## MATERIALS AND METHODS

### Cell line and animals

The B16F10 murine melanoma cell line is syngeneic in C57BL/6 mice; the YAC-1 cell line is the moloney leukemia-induced T-cell lymphoma of the A/Sn mouse origin. Both cell lines were purchased from the Cellular Institute of China in Shanghai. The cells were and was purchased from the Cellular Institute in Shanghai, China, and cultured at 37ºC in 5% CO_2_ atmosphere in RPMI 1640 supplemented with 10% calf bovine serum (Gibco BRL, USA) that contained 100U/ml penicillin G sodium and 100 μg/ml streptomycin.

C57BL/6 mice at 5 weeks of age and 16±2 gram in weight were obtained from Yang Zhou University of China (license number: SCXK, Jiangsu province of China, 2007-0001). All the mice were housed under the specified pathogen-free condition, and all experiments were performed in compliance with the guidelines by the Animal Research Ethics Board of Southeast University.

### Short hairpin RNA sequence design, construction of shRNA1 targeting TGF-β1 gene and screen of clones stably transfected with shRNA1

Short hairpin RNA (shRNA) sequence of mouse TGF-β1 was designed based on the mRNA ID (GenBank NO.NM_ 011577) using the siDESIGN design software (Dharmacon, http://www.thermoscientificbio.com/design-center/) and Block-iTTM RNAi Designer (Invitrogen, Grand island, NY) as well as BLAST (http://www.ncbi.nlm.nih.gov/ BLAST). The shRNA sequences are as follows: TGF-β1-siRNA1: sense,5′-CACCGCAACAATTCCTGGCGTTACCCGAAGGTAACGCCAGGAATTGTTGC-3′; antisense,5′-AAAAGCAACAATTCCTGGCGTTACCTTCGGGTAACGCCAGGAATTGTTGC-3′; scramble-siRNA: sense,5′-GATCCCCTTCTCCGAACGTGTCACGTTTCAAGAGAACGTGACACGTTCGGAGAATTTTTGGAAA-3′; antisense,5′-AGCTTTTCCAAAAATTCTCCGAACGTGTCACGTTCTCTTGAAACGTGACACGTTCGGAGAAGGG-3′. The primers were synthesized by Gene and Technology of China in Shanghai. A pSUPER-EGFP1 (enhanced green fluorescent protein 1) vector was used to construct the recombinant of pSUPER-EGFP1-TGF-β1-shRNA1 (shTGF-β1) as previously described [29]. A pSUPER-EGFP1-scrambled shRNA (shScramble) was used as a negative control. These recombinants were verified by the analyses of endonuclease digestion and sequencing. The B16F10 cells were transfected with either the shTGF-β1 or the shScramble by using Lipofectamine^TM^ 2000 reagent (Invitrogen, USA) following the manufacturer's protocol. After an antibiotic selection with 800 μg/ml G418 (Clontech, CA), the shTGF-β1-B16F10 clones and the shScramble-B16F10 clones were respectively isolated from G418 resistant cells for each transfection;the clones were pooled and expanded into cell lines and were named‘B16F10/shTGF-β1'and‘B16F10/Scrambled cells’ in this report. The TGF-β1 expression was detected by a Western blot [30].

### Western blot

1×10^6^ cells or tumor tissues were collected and lyzed in the protein extraction buffer (Novagen, Madison, WI, USA) by following the manufacturer's protocol. 12% sodium dodecyl sulfate polyacrylamide gel electrophoresis was performed and the proteins (10 μg/lane) were transferred onto a PVDF membrane blocked with 4% dry milk in Tris-buffered saline with Tween-20 for 1 h at 20ºC. The membrane was then incubated with the goat anti-mouse IL-21, the rabbit anti-mouse/human ZEB1 (I-18, Santa Cruz Biotechnology Company, USA), the rabbit anti-mouse/human Phospho-Smad2 /Smad3, TGF-β1, Vimentin, E-cadherin, N-cadherin, SMAD-7, (Bioworld Technology, USA), and the rabbit anti-mouse/human Gli1/Gli2 antibody, (ABcam Company, USA), respectively for overnight at 4ºC. The membrane was rinsed for 5 min with an antibody wash solution for 3 times before adding to it the goat anti-rabbit or the rabbit anti-goat fluorescence secondary antibody. Immunoreactive bands were detected by Odyssey scanning instrument (LI-COR Odyssey Imaging System, USA ) [30].

### Colony forming assay

We investigated the colony formation number in the differently treated B16F10 cells. A colony with a diameter larger than 75 μm or having more than 50 cells was counted for 1 positive colony according to our previous report [12]. The number of colonies means that the colony formation number in 100 differently treated B16F10 cells.

### Animal experiment

The C57BL/6 mice were initially immunized subcutaneous (s.c.) in a mouse's right flank with 1×10^6^ B16F10/GPI-IL-21 tumor vaccine inactivated with the 100 μg/ml^−1^ mitoxantrone; this was performed three times at a two-week interval [7]. About 14 days after the final immunization, the mice were randomly divided into three groups of equal size (6 mice per group). The three groups were the B16F10/GPI-IL-21+ B16F10/shTGF-β1 group (the mice were challenged by 5×10^5^ B16F10/shTGF-β1), the B16F10/GPI-IL-21+B16F10/shTGF-β1+ miR200c group (the mice were challenged by 5×10^5^ B16F10/shTGF-β1 accompanied with the injection of miR200c agomir), and the B16F10/GPI-IL-21+B16F10/Scrambled + miR200c/Scrambled group (the mice were challenged by 5×10^5^ B16F10/Scrambled cells accompanied with the injection of miR200c/Scrambled). The B16F10/GPI-IL-21 vaccine was developed as reported previously [8]. The agomir and the miRNA negative control were synthesized by Bioribo Company and were applied as reported previously [31]. We locally injected 1.5 nmol miR-200c agomir (micrONTM miRNA agomir mouse mir-200c-3p, Bioribo Company) in 0.1 ml saline buffer were locally injected into a B16F10 cells-forming tumor mass once every 4 days for 8 times. The non-vaccinated mice were used as the control, and the mice were challenged by 5×10^5^ B16F10/shTGF-β1(the B16F10/shTGF-β1 group)or by 5×10^5^ B16F10/shTGF-β1 accompanied with the injection of miR200c agomir (the B16F10/shTGF-β1+ miR200c group). The model of lung metastasis was set up by injection of 1×10^6^ the different treated B16F10 cells by the tail veins 14 days after the final immunization. The experiment was repeated twice. Tumor growth was monitored every three day by measuring the two perpendicular tumor diameters with calipers. The B16F10 cells-forming tumors were submitted for an immunohistochemistry and Western blot assays. The lungs, lymph nodes, kidneys, liver, and heart tissues of the mice were harvested on Day 51, and were stained with hematoxylin and eosin (H&E) for counting the number of tumor metastases [13].

### ELISA for IFN-γ and TGF-β1

The enzyme linked immunosorbent assay (ELISA) was performed to detect IFN-γ and TGF-β1 by following the Kit's protocol (eBioscience, CA). The Kit is suitable for detecting samples that include cell culture supernatant and serum; the sensitivity of Kit is 5.3 pg/Ml [19, 32].

### Measurement of splenocyte cytotoxicity

In the cytotoxic assay, the B16F10 and YAC-1 cells were used as the target cells of cytotoxic T lymphocyte (CTL) and the natural killer (NK) cells, respectively. The harvested splenocytes containing the CTL and NK cells were used as the effector cells that were labeled with 0.5 mM 5-(and 6)-carboxy-fluorescein diacetate succinimidyl ester (CFSE; 20 μg/ml) at 37°C for 15 min. The labeled splenocytes were washed twice in the phosphate-buffered saline (PBS) containing 5% calf bovine serum to sequester any free CFSE. The CFSE-labeled effector cells were seeded with a constant number of B16F10 or the YAC-1 target cells in a 96-well plate at the 25:1 ratios of the effector cells to the target cells (E:T). The cytotoxicity assay was performed in triplicate. The flow cytometric CFSE/7-AAD cytotoxicity assay was analyzed with a Flow Cytometry (FCM, BD company, USA) [33, 34].

### CD4^+^CD25^+^Foxp3^+^T cells staining and FCM analysis

Fifty one days after different B16F10 cell challenges, the inguinal lymph nodes and the splenocytes were aseptically removed, respectively, from mice and multiple lymph nodes were pooled from the different groups. The suspensions of the lymphoid cells and the splenocytes were respectively prepared by mechanical disruption with the blunt end of a 3-mL plastic syringe in PBS. Intracellular staining of the forkhead box protein P3 (Foxp 3)-expressing T cells was performed on the cells directly by the PCH101 anti-Foxp 3 antibody according to the manufacturer's protocol [35, 36]. For the analysis of CD4^+^or CD25^+^-expressing T cells, the cell suspensions were co-stained with the fluorescein isothio-cyanate (FITC)-conjugated anti-CD4^+^antibody and phycoerythrin (PE) conjugated anti-CD25^+^(eBioscience, San Diego, CA, USA). These differently conjugated cells were kept away from light for 30 min at 4°C and small lymphocytes were gated according to forward/side-scatter profiles; data were collected by using FCM within 1 h after staining, and then analyzed by using the Cell Quest software (Becton Dickinson) [37, 38].

### Immunohistochemistry

Immunostaining was performed as reported previously [13, 39]. Briefly, 5μm-thin formalin fixed and paraffin-embedded tumor slides were incubated with the rabbit anti-mouse/human TGF-β1, ZEB1, Vimentin, E-cadherin, N-cadherin, SMAD-2/3, SMAD-7 Gli1/Gli2, respectively, overnight at 4°C. The antibody concentration was 1:500. The samples were then labeled with horseradish peroxidase-conjugated streptavidin (Invitrogen) and the chromogenic reaction that was developed by using Liquid DAB Substrate Pack according to the manufacturer's instructions. The stained cells from random and non-overlapping fields were counted under a magnification of ×400.

### Statistical analysis

Statistical comparisons were performed using the Student's t-test method or single factor analysis of variance to test for any statistically significant differences in the results between the experiment group and the control group. The Bonferroni correction was applied where multiple comparisons were made. A *P* value below 0.05 was considered statistically significant.
